# Chemical reporters to study mammalian O-glycosylation

**DOI:** 10.1042/BST20200839

**Published:** 2021-04-16

**Authors:** Kathryn E. Huxley, Lianne I. Willems

**Affiliations:** York Structural Biology Laboratory, Department of Chemistry, The University of York, York YO10 5DD, U.K.

**Keywords:** chemical biology, chemical reporter, chemoenzymatic labelling, glycobiology, glycosylation, metabolic engineering

## Abstract

Glycans play essential roles in a range of cellular processes and have been shown to contribute to various pathologies. The diversity and dynamic nature of glycan structures and the complexities of glycan biosynthetic pathways make it challenging to study the roles of specific glycans in normal cellular function and disease. Chemical reporters have emerged as powerful tools to characterise glycan structures and monitor dynamic changes in glycan levels in a native context. A variety of tags can be introduced onto specific monosaccharides via the chemical modification of endogenous glycan structures or by metabolic or enzymatic incorporation of unnatural monosaccharides into cellular glycans. These chemical reporter strategies offer unique opportunities to study and manipulate glycan functions in living cells or whole organisms. In this review, we discuss recent advances in metabolic oligosaccharide engineering and chemoenzymatic glycan labelling, focusing on their application to the study of mammalian O-linked glycans. We describe current barriers to achieving glycan labelling specificity and highlight innovations that have started to pave the way to overcome these challenges.

## Introduction

Glycans play crucial roles in a diversity of cellular processes. For example, glycoproteins are essential mediators of cell–cell and cell–matrix interactions, they are involved in cell recognition and signalling events, and they control the stability, localisation and function of proteins [1,2]. Glycans have also been identified as key players in a range of pathological processes, including host–pathogen interactions [3], neurodegenerative disorders [4], and cancer metastasis [5]. The major types of vertebrate glycans are *N*-linked and O-linked glycans, which are attached to the side chain nitrogen atom of asparagine residues or the hydroxyl group of an amino acid side chain (usually serine or threonine), respectively. *N*-linked glycans consist of a universal core structure that starts with an *N*-acetylglucosamine (GlcNAc) and is further extended and edited by networks of glycosyltransferases and other glycan-modifying enzymes, resulting in a large diversity of mature *N*-glycan structures [2].

Contrastingly, O-glycans do not have a common core structure and are classified by the nature of the first monosaccharide residue that is linked to the protein. The most common type of cell surface O-glycosylation is mucin-type glycosylation, in which the first monosaccharide is an *N*-acetylgalactosamine (GalNAc) [6]. Other types of O-glycosylation include glycans initiated by GlcNAc, mannose, fucose, glucose or xylose residues [2]. A unique form of glycosylation found on nuclear, cytosolic and mitochondrial proteins of eukaryotic cells is the O-GlcNAc modification, in which a single GlcNAc residue is attached to serine or threonine hydroxyl groups. The O-GlcNAc modification is not further extended and is a highly dynamic regulator of diverse cellular processes [7].

The immense structural diversity of glycans, their non-genetically encoded nature and the complexity of glycan biosynthesis make it difficult to unravel the biological functions and structures of individual glycan species. At the same time, a cell's glycome — the total set of glycans that is present in a cell at any given time — is dynamic and will vary in response to changes in the physiological state of the cell. Tools and techniques that help us characterise glycans and modulate their function in a native cellular context offer great opportunities to advance our understanding of glycobiology and can provide avenues for therapeutic intervention. Chemical reporters enable the visualisation, enrichment and/or modulation of glycan structures by introducing unnatural tags into specific monosaccharide residues within native glycans of live cells or organisms. In some cases, the introduced tag is a fluorophore or other reporter group, but more typically, it is a small group with unique chemical reactivity that serves as a handle for further conjugation to a second reagent through what is known as ‘bioorthogonal chemistry’ [8,9]. Bioorthogonal reactions involve reagents that can react selectively and efficiently with each other in a biological environment but are inert towards functionalities present in biomolecules, a classic example being the ‘click’ cycloaddition reaction between azides and alkynes [10,11].

**Figure 1.**
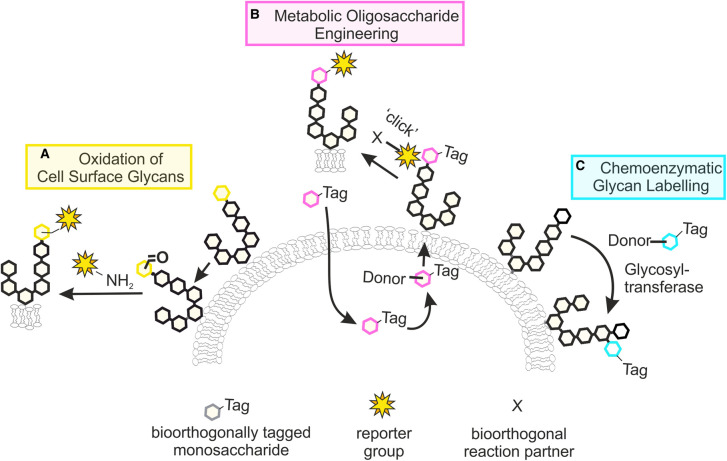
Chemical reporter strategies to study glycans. (**A**) Chemical or enzymatic oxidation of glycans enables the labelling of specific monosaccharide residues by reaction with amine-functionalised reporter groups. (**B**) Metabolic oligosaccharide engineering makes use of the cell's endogenous glycan biosynthetic machinery to install unnatural monosaccharide derivatives into glycans. The introduced tags can be further labelled by bioorthogonal ‘click’ reactions. (**C**) Chemoenzymatic glycan labelling exploits the activity of recombinant glycosyltransferases to transfer unnatural monosaccharides onto specific glycans.

Early approaches for the covalent tagging and enrichment of cell surface glycans relied on the chemical or enzymatic oxidation of specific carbohydrate residues, leading to the formation of aldehydes that can be conjugated to an amine-linked reporter group (Figure 1A) [12,13]. While these methods are still used today, they are limited to the labelling of sialic acid and galactose- or GalNAc-containing glycans and offer little flexibility in the choice of labelling chemistry. Over the past decades, various chemical reporters have been developed that enable the labelling of endogenous glycans through metabolic and chemoenzymatic glycan engineering strategies [14–19]. In these approaches, termed metabolic oligosaccharide engineering (MOE, Figure 1B) and chemoenzymatic glycan labelling (CeGL, Figure 1C), unnatural carbohydrate derivatives are incorporated into glycans by the cell's own metabolic machinery or by the action of recombinant glycosyltransferases, respectively. Advances made in these areas, in combination with a growing number of bioorthogonal ligation reactions [8,20], have greatly expanded our glycan labelling toolkit. In this mini-review, we will discuss key concepts of MOE and CeGL strategies, focusing on their application to the study of mammalian O-glycosylation, and will provide the reader with an overview of recent advances and future challenges.

## Oxidation of cell surface glycans

**Figure 2.**
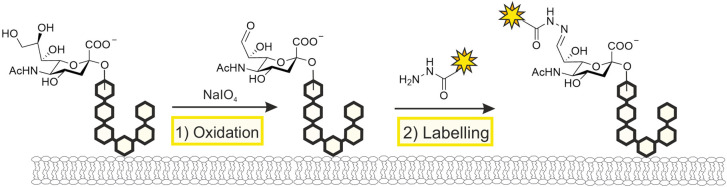
Chemical oxidation of cell surface glycans. Monosaccharides that carry a *cis* diol motif, such as sialic acids, are sensitive to oxidation by periodate treatment. The aldehyde generated upon oxidative cleavage can react with an amine nucleophile, such as hydrazide, for conjugation to a desired reporter group.

A subset of natural monosaccharides, including sialic acids and galactose, display a *cis* diol motif that is susceptible to oxidative cleavage by reagents such as sodium periodate. This unique property forms the basis of a chemical tagging strategy based on the chemical oxidation of glycans, which specifically targets monosaccharides with *cis* diol containing monosaccharides (Figure 2) [13]. The resulting aldehyde intermediates in turn provide handles for further reaction with amine nucleophiles such as hydrazine or aminooxy reagents. The utility of this approach, especially in the context of live-cell imaging, has been limited by the slow kinetics of the oxime ligation step and its optimal reaction pH of 5–6. A considerable advance was made by the Paulson group, who introduced an aniline catalyst that drastically enhances labelling efficiency at neutral pH [21]. Termed Periodate oxidation and Aniline catalysed oxime Ligation (PAL), this approach allows the labelling of glycans on the surface of living cells without affecting cell viability.

Selectivity for the labelling of certain glycan types can be achieved by careful tuning of the oxidation conditions. Mild periodate oxidation leads to the selective oxidation of cell surface sialic acids, a strategy that has been used to enrich sialylated *N*- and O-glycans [22]. Harsher oxidation conditions can be used to oxidise *trans* diols in carbohydrates such as GlcNAc. This approach permits the isolation and characterisation of O-GlcNAc modified proteins [23]. Alternatively, cell surface glycans can be oxidised by enzymatic oxidation using a specific galactose oxidase that targets terminal galactose- and GalNAc-containing glycans [12]. As for PAL, oxidation is followed by an oxime ligation step that is accelerated in the presence of an aniline catalyst [24]. Specificity for other types of monosaccharides has been achieved by the generation of engineered variants of galactose oxidase that target, for example, *N*-glycolylneuraminic acid (Neu5Gc) [25].

The main limitation of these approaches is the potential for off-target reactivity, which may occur either during the ligation step due to the presence of naturally occurring carbonyl groups, or during the periodate oxidation step through oxidation of 2-amino alcohols found in *N*-terminal serine and threonine amino acids of proteins [23]. One solution to this problem is the protection of the *N*-terminus via dimethyl labelling, which inhibits oxidation at these sites [26]. Despite these drawbacks, both chemical and enzymatic oxidation strategies are useful methods to monitor changes in glycosylation status, which are often a hallmark of disease [27]. Kohler and co-workers used both sialic acid labelling with PAL and labelling with galactose oxidase to identify host glycoproteins that are desialylated by pneumococcal neuraminidases [28]. Neuraminidase substrates were identified by detecting either a loss in PAL-mediated labelling or a gain in galactose oxidase-mediated labelling due to the exposure of galactose residues upon the loss of sialic acids.

## Metabolic oligosaccharide engineering

**Figure 3.**
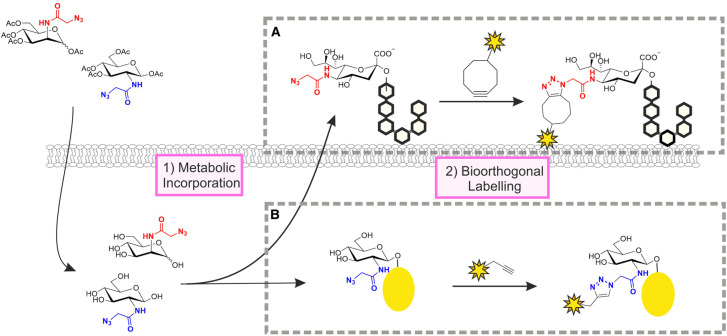
Metabolic oligosaccharide engineering. Cells are treated with unnatural, tagged derivatives of naturally occurring monosaccharides, such as the peracetylated azide-tagged derivatives of ManNAc and GlcNAc shown (top left). After uptake and deprotection of the acetyl groups (bottom left), the metabolic precursors enter the cellular pathways for conversion into the corresponding CMP- or UDP-activated donors, which are then used for glycan biosynthesis. ManNAc derivatives are incorporated as sialic acids into cell surface glycans (**A**) while GlcNAc derivatives label intracellular proteins that are targets for O-GlcNAc modification (**B**). The azide-tagged glycans can be labelled by bioorthogonal ligation reactions such as the strain-promoted (**A**) or the copper(I)-catalysed (**B**) azide-alkyne cycloaddition.

As an alternative to the direct chemical modification of native glycan structures, MOE exploits the flexibility in the cell's own metabolic machinery for the introduction of tagged monosaccharides into desired glycans (Figure 3). This approach was pioneered by Reutter and co-workers who showed that cells fed with unnatural derivatives of *N*-acetylmannosamine (ManNAc) are able to convert them into the corresponding cytidine monophosphate (CMP)-sialic acid donors and incorporate the unnatural substrates into cell surface glycans [29]. Although their strategy was not used to install tags for detection, it demonstrated that the cellular machinery for glycan biosynthesis tolerates structurally modified analogues of ManNAc and sialic acid with larger *N*-acyl substituents than their natural counterparts. The Bertozzi group then showed that the same principle can be applied to the introduction of ketone- or azide-tagged sialic acids into cell-surface glycans (the azide strategy is shown in Figure 3A) [30,31]. The azide is one of the most popular bioorthogonal tags and is typically labelled through azide-alkyne cycloadditions, which are either catalysed by a copper(I) catalyst (Figure 3B) or accelerated by the relief of ring strain from a cyclooctyne reagent (Figure 3A) [32,33]. Following these initial reports, the scope of MOE has expanded to include derivatives of a wide variety of different monosaccharides and diverse types of bioorthogonal tags, which cannot all be covered here. Instead, for comprehensive overviews of the various chemical reporters and bioorthogonal chemistries that have been developed to date, we direct the readers to recent reviews [8,9,15–20].

One of the key considerations for the design and evaluation of MOE probes is glycan specificity. Since most monosaccharides enter multiple glycan biosynthetic pathways, tagged derivatives of these carbohydrates will similarly be incorporated into various glycan structures. For example, while *N*-azidoacetylglucosamine (GlcNAz) labels primarily intracellular proteins that are O-GlcNAc modified (Figure 3B) [34], it can also be installed in both *N*-glycans and mucin type O-glycans on the cell surface [35,36]. A study by Boyce et al. revealed that the *N*-acetylgalactosamine derivative GalNAz provides access to O-GlcNAc modified proteins through an endogenous cellular pathway that interconverts uridine diphosphate (UDP)-GlcNAc and UDP-GalNAc donors [37]. While this discovery made it possible to label O-GlcNAcylated proteins with higher efficiency than that observed for GlcNAz itself, it also highlights the potential complication of MOE experiments due to crosstalk between metabolic pathways.

The type of bioorthogonal tag and its position on a monosaccharide can have an impact on both the incorporation efficiency of chemical reporters and their selectivity for certain glycan types [36,38,39]. Pratt and co-workers demonstrated the dramatic effects that an apparently small change in tag, going from an azide to an alkyne, can have on the overall labelling efficiency of MOE as well as on the set of glycans that is labelled [36]. This effect was shown to be dependent on cell type and the type of monosaccharide used. While initial MOE approaches relied on attachment of the unnatural tag at the *N*-acyl group of hexosamines, several more recent studies have explored the substitution of hydroxyl groups [40–44] or the *N*-acyl group in GlcNAc [45] with a bioorthogonal tag. In many cases, this strategy has led to the development of chemical reporters with enhanced selectivity towards specific classes of glycans. For instance, 6-azido-6-deoxy-GlcNAc is selectively installed on O-GlcNAc modified proteins but not cell surface glycans [40]. Strikingly, selectivity within a glycan class was reported for a neuraminic acid derivative carrying a sydnone ligation handle at C9 [42]. In contrast with its C9-azide-tagged analogue, the sydnone probe selectively labels a subpopulation of sialylated glycoproteins in a glycosidic linkage-specific manner.

The overall success of MOE approaches relies on both the efficiency of unnatural carbohydrate incorporation into glycans and the efficiency of the ensuing bioorthogonal labelling reaction. Comparative studies by Dold et al. revealed that incorporation efficiency can vary widely, even for structurally closely related probes, from <1% to more than 60% [46,47]. Advances in bioorthogonal ligation kinetics have been shown to enable efficient glycan labelling even if incorporation rates of the MOE probes are as low as 1% [48]. At the same time, other studies have demonstrated that improved reaction kinetics do not always lead to improvements in glycan labelling, suggesting that the efficiency of metabolic conversion is equally important for the success of MOE probes [49]. Quantification of cellular levels of monosaccharides and the corresponding nucleotide-activated glycosyl donors can provide additional insights into the effects of specific structural changes on the efficiency with which unnatural carbohydrate derivatives are taken up and metabolised by cells [46,50,51].

The hydroxyl groups in chemical reporters are often protected with acetyl groups to reduce polarity and thereby enhance cell permeability of the molecules. In a cautionary report, however, Chen and co-workers demonstrated that peracetylated monosaccharides cause non-enzymatic labelling of cysteine residues, which leads to misidentification of endogenous glycosylation sites [52]. It was later reported that the extent of S-glycosylation is dependent on the reporter used [53] and that partial protection of azide-tagged *N*-acyl hexosamines at the C1 and C3 positions alleviates the problem [54]. It has also been shown that acetyl groups can enhance the cytotoxicity of chemical reporters, especially when installed at C6 [45,55], although these effects appear to depend on other structural properties of the reporters as well [39,56]. In light of the potentially harmful properties of acetyl groups, it is interesting to note that not all chemical reporters will need to cross the cell membrane. Gilormini et al. studied the cellular uptake mechanisms of unprotected alkyne-tagged ManNAc and sialic acid derivatives and concluded that these are internalised by an as yet unidentified transporter and endocytosis, respectively [57]. A GLUT1 transporter was shown to be responsible for the cellular uptake of peracetylated galactose-derived probes [43].

Moving beyond the metabolic labelling of single types of monosaccharides, several groups have explored combinations of multiple bioorthogonal ligations for the simultaneous labelling of two glycan populations on the same cells [48,56,58]. Building on the latest advances in bioorthogonal chemistry, a triple labelling of cell surface glycans using ManNAc derivatives with three different tags has also been described [59]. The Vocadlo group used a dual bioorthogonal strategy to verify the existence of co-translational O-GlcNAcylation, a modification that had thus far been believed to occur only post-translationally [60]. Two other innovations in MOE-based glycan labelling involved the imaging of protein-specific O-GlcNAcylation [61] and the use of a directly fluorescently labelled MOE probe [62]. Doll et al. used FLIM-FRET imaging to monitor the glycosylation status of individual proteins in living cells by combining genetically encoded fluorescent tags (EGFP) with a chemical reporter for O-GlcNAc that was reacted with a cell-permeable fluorophore [61]. Vocadlo and co-workers developed a fluorescently labelled metabolic precursor for UDP-GlcNAc that is tolerated by O-GlcNAc transferase (OGT) and allows the direct visualisation of O-GlcNAcylated proteins in cells without the need for bioorthogonal ligation [62]. Approaches such as these hold great potential for studying the dynamics of protein-specific glycosylation and glycosyltransferase activity in living cells.

The developments in MOE made over the past decade have made a great impact on our ability to visualise, characterise and/or modulate glycan structures in live cells and even in whole organisms [15,63,64]. Even though exogenous monosaccharides can impact metabolic pathways and glycan structures [65], the direct incorporation of tags into endogenous glycans nonetheless provides great opportunities for monitoring cellular glycans and dynamic changes that occur, for example, during cell maturation and development [33,44,66]. A key challenge remains labelling selectivity, which is limited by the incorporation of monosaccharides into multiple glycan types and the cellular interconversion of monosaccharide metabolites. Recent advances in the targeted delivery of MOE probes enable the labelling or engineering of glycans on specific cell types only. Examples include the use of liposomes for targeted delivery [41,67] and methods that exploit enzymatic ‘uncaging’ of inactive MOE precursors at a target site [68]. Such cell type specific approaches can in turn serve as a targeting strategy for the selective delivery of nanoparticles carrying, for example, imaging agents or therapeutics [69,70].

## Chemoenzymatic glycan labelling

To address the glycan specificity issues of MOE, an alternative labelling strategy has been developed termed chemoenzymatic glycan labelling (CeGL) [18]. This method exploits the specialised activity of specific recombinant glycosyltransferases to transfer a modified monosaccharide analogue from a suitable glycosyl donor onto a specific glycan acceptor. First described in 1979 for the transfer of radiolabelled [^14^C]-sialic acid onto cell surface glycans [71], the field of CeGL has since grown in parallel to the fields of MOE and bioorthogonal chemistry. It now encompasses many different bioorthogonal tags and reporter groups and offers the ability to target a variety of monosaccharide acceptor and donor specificities [18,64,72].

**Figure 4.**
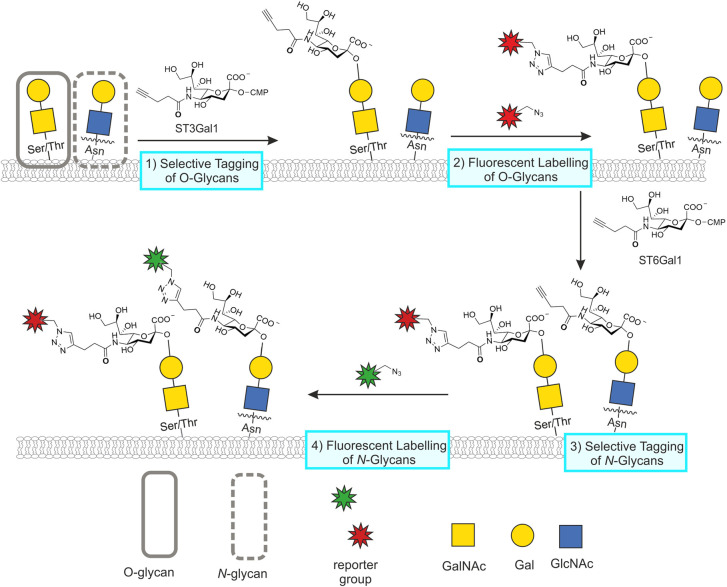
Tandem chemoenzymatic glycan engineering. In CeGL, recombinant glycosyltransferases are used to transfer unnatural monosaccharides from appropriate donors onto glycan acceptors. Wu and coworkers [73] designed a double labelling strategy with successive reactions catalysed by ST3Gal1 and ST6Gal1, installing the same alkyne-tagged CMP-sialic acid derivative onto unextended Gal-GalNAc disaccharides present on O-glycans or uncapped Gal-GlcNAc disaccharides at the non-reducing end of *N*-glycans, respectively. Tagged glycans were labelled via two separate azide-alkyne cycloaddition reactions for the simultaneous visualisation of both glycan types at the cell surface with different fluorophores.

CeGL facilitates the selective targeting of *N*- or O-glycans by exploiting the substrate specificity of the sialyltransferases ST6Gal1 and ST3Gal1, respectively [73–76]. The approach was first demonstrated in 2013 for the specific labelling of *N*-glycans with C9 azido-tagged CMP-*N*-acetylneuraminic acid (CMP-Neu5Ac9N_3_) by ST6Gal1 [77]. In a comparative study by Yu et al. O- and *N*-glycans were labelled with either MOE using the chemical reporter peracetylated N-azidoacetylmannosamine (Ac_4_ManNAz) or CeGL (refered to as SEEL) using ST3Gal1 and ST6Gal1 [76]. Metabolic labelling occurred almost exclusively in O*-*glycans, while the spectral count from MS analysis was significantly higher for CeGL tagged glycans. Building on the selective labelling profiles of ST3Gal1 and ST6Gal1, Wu and co-workers utilised a double labelling approach with both enzymes in their study of human cancers [73]. This approach allowed differential visualisation of both *N*- and O-glycans in the same tissue by utilising successive labelling with ST3Gal1 and ST6Gal1. While both enzymes transferred an alkyne-tagged CMP-sialic acid donor onto their target glycans, differential fluorescent labelling was achieved through two sequential ligation steps (Figure 4).

In addition to specificity towards the broader classes of O- and *N*- glycans, selectivity of the CeGL methodology has been narrowed down to the identification and labelling of specific glycan structures. In 2011, Zheng et al. developed an effective method for labelling *N*-acetyllactosamine (LacNAc), a galactose(Gal)-β1,4-GlcNAc disaccharide, by utilising a recombinant *H. pylori* α(1,3)fucosyltransferase [78]. Other groups have applied similar strategies, using azido and alkynyl derivatives of GalNAc for selective labelling of Galβ1,3-GalNAc disaccharides also known as the Thomsen−Friedenreich antigen [79], fucose(Fuc)-α1,2-Gal glycan biomarkers [80] and the Neu5Ac−α2,3-Gal linkage [81]. Furthermore, Zhu and co-workers developed a strategy to distinguish between α2,3- and α2,6-linkages within Neu5Ac-Gal disaccharides [82].

Another attractive aspect of CeGL is the ability to employ enzymes with higher tolerance towards donor modifications. This allows for incorporation of larger reporters than those generally used for MOE, where structural flexibility is restricted by substrate tolerance of the cell's endogenous metabolic machinery. The direct incorporation of fluorophores or affinity tags has been reported to improve the efficiency of cell surface labelling of glycoconjugates compared with both two-step CeGL and MOE [83]. One-step CeGL with fluorescent donors has vastly grown in popularity and scope. Recent applications include the labelling of fucosyltransferase substrates with multiple fluorescent Fuc derivatives [84], O-GlcNAc modified proteins via a tandem labelling strategy with a fluorescent sialic acid derivative [85], and terminal galactose on *N*- and O-glycans with BODIPY-tagged sialic acid derivatives [86]. These studies reveal efficient labelling that demonstrates the high tolerance of glycosyltransferases to functional group modification, as well as the ability of CeGL to allow for direct functionalisation of target glycans.

With the aim of further advancing the scope of CeGL, Wen et al. chemoenzymatically synthesised a library of UDP-GlcNAc and UDP-GalNAc derivatives, which they screened against multiple enzymes reported to label Fucα1,2-Gal and Neu5Acα2,3-Gal epitopes [87]. The new derivatives covered a range of functionalities that broadened the scope of glycosyl donor structures. Additionally, Hong et al. have recently screened for bacterial glycosyltransferases able to directly incorporate Cy3-fluorophore and Biotin containing sugars [88]. These additions to the CeGL toolkit will facilitate further discoveries in this field.

## Bump-and-hole strategies

Bump-and-hole methodology is an approach that relies on the engineering of enzyme active sites, forming a ‘hole’ able to accommodate unnaturally modified, or ‘bumped’ substrates. The power of this approach to augment the scope of MOE was demonstrated by Yu et al. in a strategy designed to overcome the cell's poor tolerance towards a photocrosslinker-tagged GlcNAc derivative [89]. Though technically not considered true bump-and-hole, this work demonstrated that overexpression of a mutant of AGX1, the enzyme responsible for the final step in the biosynthesis of UDP-GlcNAc, led to successful metabolic labelling of O-GlcNAcylated proteins [89]. Specific engineering of glycosyltransferases to accommodate non-natural, bioorthogonally tagged sugars was first reported by Qasba and Ramakrishnan in 2002 [90]. Over the past years, bump-and-hole strategies have been used to label glycans in a cellular context. Bertozzi and co-workers used bump-and-hole methodology for the labelling of cellular glycans with engineered GalNAc transferases that accept bumped GalNAc donors [91,92]. Following on from this work, Debets et al. were able to develop a new metabolic labelling probe (GalNAzMe) that selectively labels mucin type O-linked glycans when combined with an appropriate GalNAc transferase, but is not converted into GlcNAc and therefore leads to high specificity in O-glycan labelling [93]. Though promising results have been obtained by ‘bump-and-hole’, some challenges to this method remain, including the stable expression of mutant enzymes in cells and the delivery of modified sugar nucleotides across the cell membrane [92]. With ‘bump-and-hole’ techniques for glycosyltransferases in their infancy, there is certainly potential for this method to expand the scope of chemical reporter strategies for glycan labelling [94,95].

## Perspectives

- **Importance of the field.** Chemical reporters offer unique tools to study and manipulate specific glycans in the context of a living cell or organism. The ability to covalently add a variety of tags onto native glycans enables the development of strategies that help unravel the intricacies of glycan structures and glycan dynamics and provide potential avenues for therapeutic intervention.
- **Summary of current thinking.** Great strides have been made in expanding the scope of MOE and CeGL strategies. Major challenges that remain are the selective labelling of subsets of glycans and the targeting of glycans in a protein- and/or cell type-specific manner.
- **Future directions.** Future research will be aimed at enhancing labelling specificity by clever design of chemical reporters, combined with the enhanced substrate flexibility achievable with chemoenzymatic and bump-and-hole labelling strategies. Advances in bioorthogonal chemistry will further aid in developing the necessary efficiency and selectivity of ligation reactions required to monitor low levels of glycans and their dynamics.

## Open Access

Open access for this article was enabled by the participation of University of York in an all-inclusive *Read & Publish* pilot with Portland Press and the Biochemical Society under a transformative agreement with JISC.

## Abbreviations

BODIPY: 4,4-difluoro-4-bora-3a,4a-diaza-s-indacene
CeGL: chemoenzymatic glycan labelling
CMP: cytidine monophosphate
CMP-Neu5Ac9N_3_: C9-azide tagged CMP-*N*-acetylneuraminic acid
DFGL: direct fluorescent glycan labelling
Fuc: fucose
Gal: galactose
GalNAc: *N*-acetylgalactosamine
GalNAz: *N*-azidoacetylgalactosamine
GlcA: glucuronic acid
GlcNAc: *N*-acetylglucosamine
GlcNAz: N-azidoacetylglucosamine
LacNAc: *N*-acetyllactosamine
ManNAc: N-acetylmannosamine
ManNAz: *N*-azidoacetylmannosamine
MOE: metabolic oligosaccharide engineering
MS: mass spectrometry
Neu5Ac: *N*-acetylneuraminic acid
Neu5Gc: *N*-glycolylneuraminic acid
PAL: periodate oxidation and aniline catalysed oxime ligation
UDP: uridine diphosphate
